# Mechanism of THBS1 Regulation of MDCK Cell Proliferation and Apoptosis Through TGF-β/Smad Signalling

**DOI:** 10.3390/ijms26010395

**Published:** 2025-01-04

**Authors:** Rui Li, Fan Zhang, Lijin Wang, Siya Wang, Manlin Zhou, Jun Wang, Yiyang Zhang, Xiao Tan, Weiji Chen, Kun Yang, Zilin Qiao

**Affiliations:** 1 Engineering Research Center of Key Technology and Industrialization of Cell-Based Vaccine, Ministry of Education, Lanzhou 730030, China; lirui@gsau.edu.cn (R.L.); 15174462439@163.com (F.Z.); wsy19991216@163.com (S.W.); 15888533443@163.com (M.Z.); zyy15890182033@163.com (Y.Z.); qiaozilin@xbmu.edu.cn (Z.Q.); 2 Gansu Tech Innovation Center of Animal Cell, Biomedical Research Center, Northwest Minzu University, Lanzhou 730030, China; 3 Key Laboratory of Biotechnology & Bioengineering of State Ethnic Affairs Commission, Biomedical Research Center, Northwest Minzu University, Lanzhou 730030, China; 4 China-Malaysia National Joint Laboratory, Biomedical Research Center, Northwest Minzu University, Lanzhou 730030, China; 5 School of Life Sciences and Engineering, Northwest Minzu University, Lanzhou 730030, Chinawangjun200005@126.com (J.W.); 18693259358@163.com (W.C.)

**Keywords:** MDCK cells, *THBS1*, proliferation, apoptosis, *TGF-β* pathway

## Abstract

Madin–Darby Canine Kidney (MDCK) cells are a key cell line for influenza vaccine production, due to their high viral yield and low mutation resistance. In our laboratory, we established a tertiary cell bank (called M60) using a standard MDCK cell line imported from American Type Culture Collection (ATCC) in the USA. Due to their controversial tumourigenicity, we domesticated non-tumourigenic MDCK cells (named CL23) for influenza vaccine production via monoclonal screening in the early stage of this study, and the screened CL23 cells were characterised based on their low proliferative capacity, which had certain limitations in terms of expanding their production during cell resuscitation. It was thus our objective to enhance the proliferation efficiency of MDCK cells for influenza vaccine production following cell resuscitation, with a view to improving the production of non-tumourigenic MDCK cells for vaccines and enhancing the production of influenza virus lysate vaccines from MDCK cells through genetic intervention. We concentrated on the protein thrombosponin-1 (*THBS1*), which was markedly differentiated in the proteomics data of the two MDCK cells. By integrating this finding with related studies, we were able to ascertain that *THBS1* exerts a significant influence on the level of cell proliferation and apoptosis. Consequently, our objective was to investigate the impact of *THBS1* expression on MDCK cell apoptosis by verifying the differences in *THBS1* expression between the two MDCK cells and by interfering with *THBS1* expression in the MDCK cells. We found that the knockdown of *THBS1* significantly increased the proliferation and apoptosis of CL23 cells without causing significant changes in cell migration and invasion, and its overexpression significantly decreased the proliferation of M60 cells and increased cell migration, invasion, and apoptosis. In addition, the *TGF-β/Smad* pathway target genes transforming growth factor-β1 (*TGF-β1*), mothers against decapentaplegic homolog 2 (*Smad2*), and mothers against decapentaplegic homolog 3 (*Smad3*), were significantly down-regulated in CL23 cells after *THBS1* knockdown and up-regulated in M60 cells after overexpression, with consistent expression identified at both the mRNA and protein levels. The treatment of cells with *TGF-β* activators and inhibitors further demonstrated that *THBS1* regulated MDCK cell proliferation and apoptosis through the *TGF-β/Smad* signalling pathway. Finally, we found that *THBS1* also regulated H1N1 influenza virus replication. These findings enable a comprehensive understanding of the regulatory mechanisms of *THBS1* regarding MDCK cell proliferation and apoptosis functions and the effects of influenza virus replication.

## 1. Introduction

Influenza is a global acute respiratory disease caused by the influenza A and B viruses, which seriously affects human health and causes a disease burden due to its high morbidity and mortality. Vaccination is currently the most effective means of preventing influenza virus infection, and relevant vaccines and antiviral drugs have been developed to prevent influenza [1,2]. Traditional influenza vaccines are mainly produced using chicken embryos, but the production of such influenza vaccines based on chicken embryos cannot meet the surge in demand during a pandemic [3]. Secondly, producing influenza vaccines using chicken embryos has many limitations compared to using Madin–Darby Canine Kidney (MDCK) cells for production, such as a long supply time, a cumbersome operation process, susceptibility to contamination, and the possibility of mutation during embryonic adaptation [4]. MDCK cells effectively support the proliferation of most influenza A viruses (IVAs), and they are therefore widely used in influenza vaccine production [5].

However, because of the MDCK cells’ notable changes in cell shape, proliferation rate, and virus sensitivity, cell safety restricted its application in large-scale vaccine production [6]. A considerable number of laboratories have already adopted the use of subcellular lines as a means of meeting their influenza vaccine production requirements. Notable examples include Novartis and MedImmune, which have acquired MDCK cell lines with their own intellectual property rights and have successfully demonstrated their ability to produce influenza vaccines through the processes of cell subcloning and screening [7]. To produce a high-titer virus vaccine, increasing the cell proliferation rate for the achievement of a high cell density vaccine production process is also an important research focus. Direct application of wild-type epidemic strains for vaccine production in a tertiary biosafety environment is an important reason why the proliferation rate of MDCK cells used to produce influenza vaccines needs to be increased [8]. Accordingly, an investigation into the means of accelerating the proliferation rate of MDCK cells for the production of influenza vaccines constituted the core objective of our research programme. At present, research into cell growth and proliferation is primarily focussed on the cell cycle and metabolic regulation. However, the success rate in achieving high cell densities to develop vaccine production processes remains relatively low. It is therefore imperative to develop alternative cultivation methods for MDCK cell lines, such as genetic engineering techniques, in order to achieve a higher cell proliferation rate.

Accordingly, the protein thrombosponin-1 (*THBS1*), which is associated with proliferation and apoptosis, was identified on the basis of previous MDCK cell proteomics data profiling. *THBS1* can stimulate or inhibit cell adhesion, proliferation, and migration in an environment-dependent and cell-specific manner, but *THBS1*’s hydrolysed and recombinant N-terminal protein portion has significant pro-angiogenic activity, mediated primarily by β-1 integrins, whereas the interaction of 3TSR with α5β1 integrins inhibits endothelial cell migration in a *PI3K*-dependent manner [9]. The *TSP-1 TSR* also interacts with *CD148*, a transmembrane protein tyrosine phosphatase expressed in endothelial cells, thereby inhibiting endothelial cell proliferation and angiogenesis [10]. *THBS1* is a 450 kDa single-subunit homodimeric matrix glycoprotein and the first recognised member of the *THBS* family, consisting of N- and C-terminal globular domains linked by a pre-collagen homology region, type I alkyrin repeat, type II epidermal growth factor repeat, and type III calcium-binding region repeat. It plays a multifunctional role in cell–matrix and cell–cell interactions, as well as angiogenesis and tumours [5,11,12]. *THBS1* is secreted by many types of cells in response to injury or specific cytokines. It is transiently present in the extracellular matrix, but is rapidly internalised and degraded by fibroblasts and endothelial cells; however, *THBS1* is abundant in megakaryocytes and platelets and constitutively expressed in the dermal–epidermal border of the skin and the subendothelial matrices of some blood vessels [13]. *THBS1* can participate in important physiological processes, such as embryonic development, neovascularisation, and tissue repair, but it is also closely associated with the development of many diseases, especially tumours [14]. Most, but not all malignant tumour cells exhibit down-regulated *THBS1* expression during malignant progression [15], which is due to attenuated positive *THBS1* gene regulation by the oncogenes *TP53* and *NMEI*, as well as the enhanced negative regulation of oncogenes such as *RAS* and *MYC*. Secondly, *THBS1* expression is induced by *TGF-β*, vitamin A, progesterone, and analogues, but inhibited by *IDI* and *HGF* (hepatocyte growth factor) [16].

Reduced *THBS1* levels are also associated with poor prognosis in a variety of cancers, including non-small cell lung cancer [17], pancreatic adenocarcinoma, gastric cancer [18], invasive cervical cancer, and oral squamous cell carcinoma [19], whereas *THBS1* is positively correlated with infiltration in hepatocellular carcinoma [20]. *THBS1* also inhibits cell adhesion, cell growth, and cell motility [21], and induces apoptosis in endothelial cells and several other cell types [22]. Through screening the signalling pathways regulated by *FGF7/FGFR2*-driven *THBS1* expression, the key pathways for fibroblast growth factor signalling were identified as the *Ras–MAP* kinase pathway, involving *ERK1/2*, *p38* and *JNK* kinases, and the *PI3K–Akt* pathway, and using a range of inhibitors, it was demonstrated that *FGF7/FGFR2*-mediated *THBS1* up-regulation may occur through the *PI3K/Akt/mTOR* pathway [23]. *THBS1* expression is regulated by the *Akt* and *PI3K* pathways, as well as several growth factors, such as *P53*, *ras*, *c-src*, transforming growth factor-β, and fibroblast growth factor-2 [24]. *IGFBP3*, a functional secreted extracellular regulator protein, regulates *THBS1* via *THBS1* promoter expression, mainly through the intracellular pathway of *IGFBP3* [25]. In addition, Wang, Y et al. [26] reported that *THBS1* is a potential target gene of *METTL14* that functions as a tumour suppressor in prostate cancer (PCa), demonstrating that *METTL14* inhibits *THBS1* expression in the nucleus in an m6A-dependent manner and promotes PCa proliferation. However, the precise function of *THBS1* in controlling the phenotypes of MDCK cells, including proliferation and apoptosis, remains unclear. In addition, the production of a vaccine by knocking down *THBS1* has not been reported in the literature to date. However, Cui DL et al. [27] reported that the *THBS1* gene may be associated with the prevention and treatment of COVID-19. Consequently, our objective is to develop high-proliferative MDCK cell lines for vaccine production through the genetic engineering of MDCK cells. In this study, MDCK cell lines with *THBS1* knockdown or overexpression were constructed to explore the effects of *THBS1* on the proliferation and apoptosis of MDCK cells, and the mechanisms underlying these effects.

## 2. Results

### 2.1. Proliferation- and Apoptosis-Related Gene Detection

**Figure 1:**
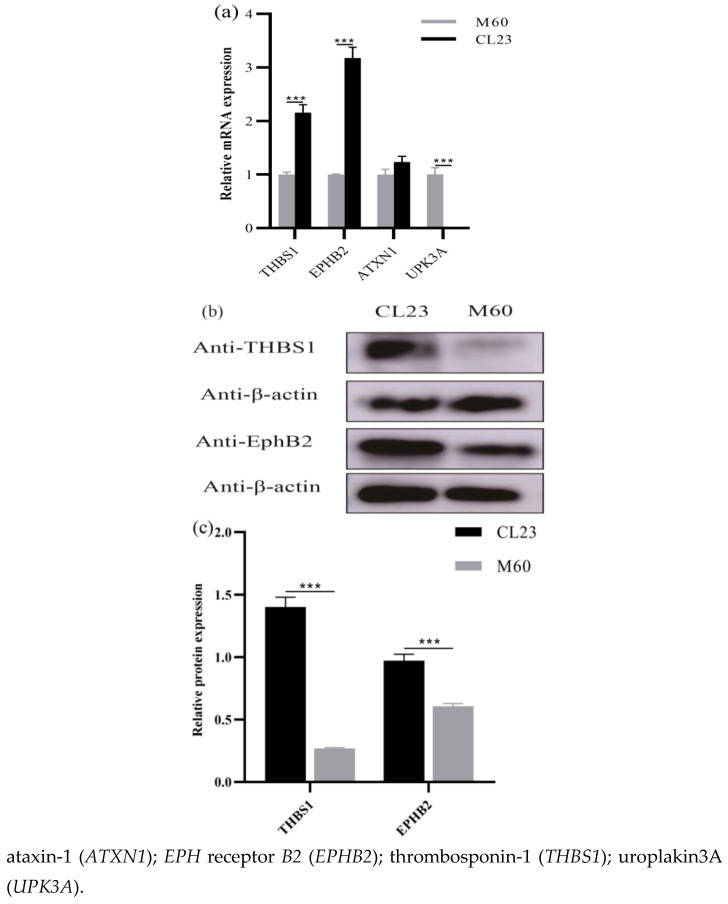
Proliferation- and apoptosis-related gene detection in CL23 cells and M60 cells. (**a**) Proliferation- and apoptosis-related genes were expressed at the mRNA level in CL23 cells and M60 cells. (**b**) Proliferation- and apopto sis-related genes were expressed at the protein level in CL23 cells and M60 cells. (**c**) *THBS1* and *EPHB2* protein level expression in CL23 cells and tumourigenic M60 cells. Differential grey value analysis. * indicates statistically significant difference (*** *p* < 0.001) and no * indicates no difference.

We verified the differences in the expression of proliferation- and apoptosis-related genes in MDCK cells by showing that *THBS1* and *EPHB2* were highly expressed at the mRNA level in CL23 cells, while *UPK3A* was poorly expressed in CL23 cells, and the difference in *ATXN1* expression between CL23 and M60 cells was not significant (Figure 1a). Moreover, the *THBS1* and *EPHB2* proteins were highly expressed in CL23 cells (Figure 1b,c). Given that *THBS1* is associated with neoangiogenesis, tissue repair, and functions such as endothelial cell proliferation and migration, we postulated that *THBS1* might be related to the regulation of proliferation and apoptosis in MDCK cells. Furthermore, we screened *THBS1* and *EPHB2* through the preliminary MDCK cell proteomics data and detected their expression differences in the two kinds of MDCK cells using RT-qPCR technology. The expression differences were more significant at the Western blot protein level for *THBS1*, and thus it was determined that the *THBS1* protein was selected for further investigation into its potential role in regulating proliferation and apoptosis in MDCK cells.

### 2.2. Determination of the Proliferation, Apoptosis, and Migration Capacity of M60 and CL23 Cells

**Figure 2:**
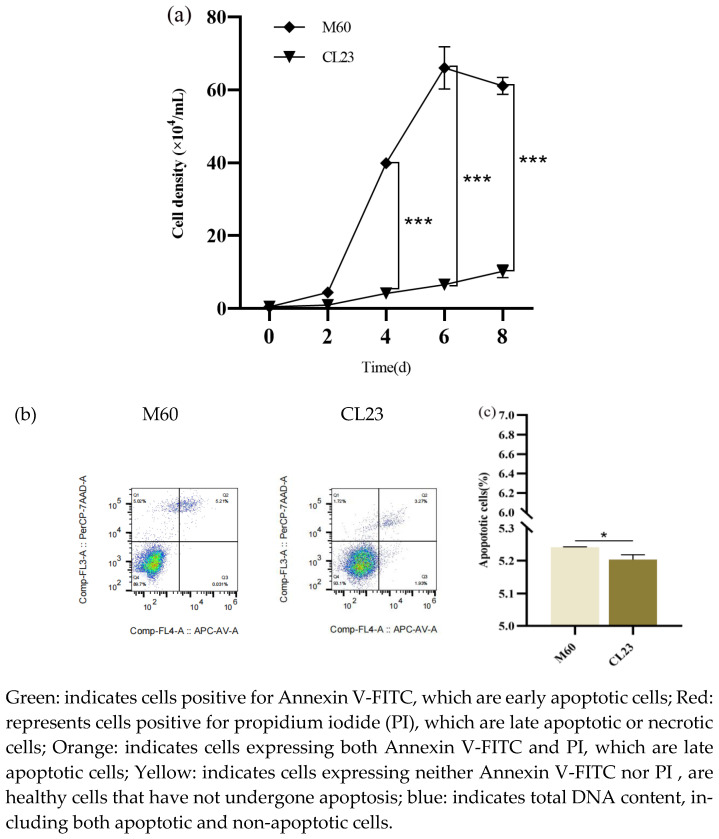

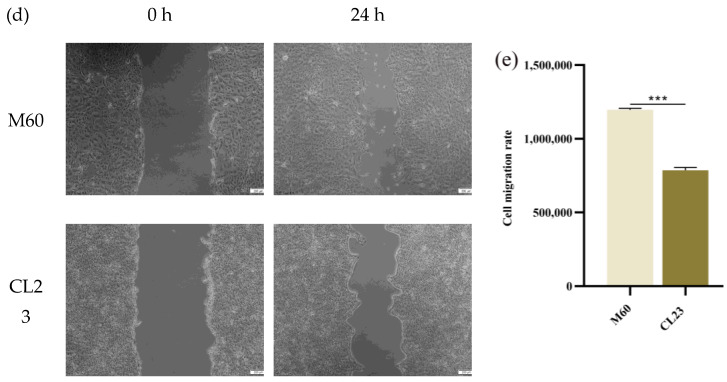
Analysis of proliferation, apoptosis, and migration ability of M60 cells and CL23 cells. (**a**) Growth curves of M60 cells and CL23 cells. (**b**,**c**) Levels of apoptosis of M60 cells and CL23 cells. (**d**,**e**) Levels of migration of M60 cells and CL23 cells. * indicates a statistically significant difference between tumour-free MDCK cells and tumour-forming MDCK cells (* *p* < 0.05; *** *p* < 0.001) and no * indicates no difference.

To determine the differences in the proliferation, apoptosis, and migration abilities of M60 and CL23 cells, the cells’ growth curves and apoptosis and migration abilities were determined in this study. The results show that M60 and CL23 cells were in the latent phase on days 0–2, entered the logarithmic growth phase on days 2–4, and entered the plateau phase on day 6. The difference in the proliferation rates of cells in the latent phase on days 0–2 was not significant, and the proliferation rate of M60 cells was significantly higher than that of CL23 cells on days 4–8 (*p* < 0.001) (Figure 2a). Apoptosis detection via flow cytometry showed that the apoptotic capacity of the M60 cells was higher than that of the CL23 cells at stage two, and the apoptotic capacity of the M60 cells was lower than that of the CL23 cells at stage three. Moreover, the total apoptotic capacity of the M60 cells was significantly higher than that of the CL23 cells (*p* < 0.05) (Figure 2b,c). The migration ability, as detected through cell scratching, was significantly higher for M60 cells than CL23 cells within a 0–24 h period (*p* < 0.001) (Figure 2d,e).

### 2.3. Construction and Characterisation of THBS1-Knockdown and -Overexpressing MDCK Cell Lines

**Figure 3:**
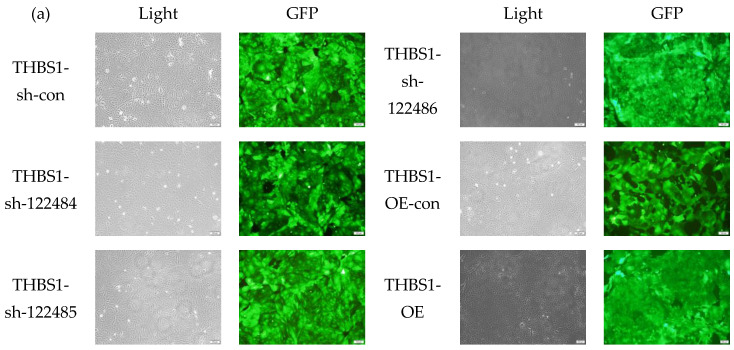

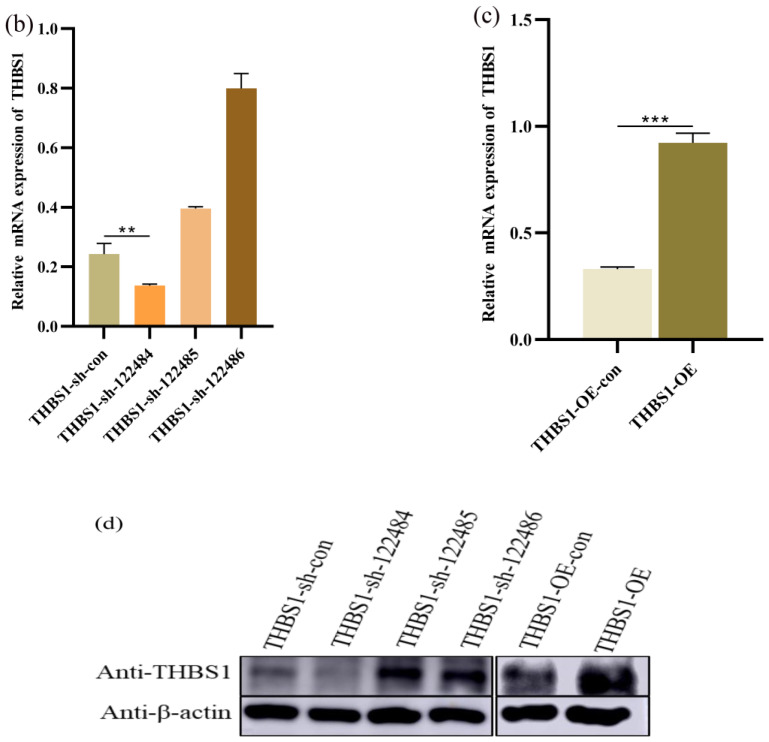

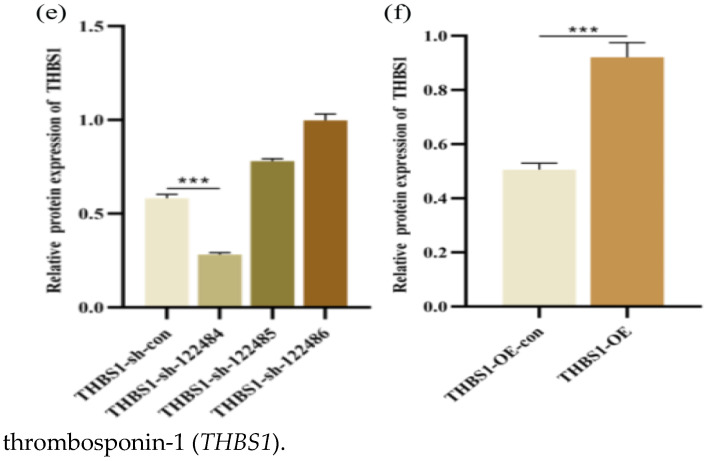
Construction and characterisation of MDCK cell lines stably knocking down and overexpressing *THBS1*. (**a**) Bright field and fluorescence expression of cells after puromycin screening of knockdown control cells *THBS1*-sh-con puromycin; bright field and fluorescence expression of cells after puromycin screening of knockdown cells *THBS1*-sh-122484 locus; knockdown cells THBS1-sh-122485 locus puromycin screened for cell bright field and fluorescence expression; knockdown cells *THBS1*-sh-122486 locus puromycin screened for cell bright field and fluorescence expression; overexpression of control cells *THBS1*-OE-con puromycin screened for cell bright field and fluorescence expression; overexpression of *THBS1*-OE cells’ bright field and fluorescence expression after puromycin screening. (**b**) Differential expression of *THBS1* mRNA levels in stable knockdown *THBS1* cells. (**c**) Differential expression of *THBS1* mRNA levels in stable overexpression *THBS1* cells. (**d**) Differential expression of *THBS1* protein levels in stable knockdown and overexpression *THBS1* cells. (**e**,**f**) Differential expression of *THBS1* protein levels in stable knockdown and overexpression cells. Grey scale value analysis of *THBS1* protein level expression differences in *THBS1* cells. * indicates statistically significant difference (** *p* < 0.01; *** *p* < 0.001) and no * indicates no difference.

The results show that the fluorescent expression of *THBS1* in stably under- and overexpressing cells after puromycin screening was above 95% (Figure 3a), proving that the knockdown and overexpression transfections were successful. The *THBS1* expression levels were detected using RT-qPCR and Western blotting, and the results show that, compared with the knockdown control cells THBS1-sh-con, the knockdown level of the *THBS1*-sh-122484 locus differed significantly, and the expression was consistent at the mRNA and protein levels (Figure 3b,d,e). Moreover, compared with the overexpressing *THBS1*-OE-con control cells, *THBS1*-OE cells demonstrated overexpression at significantly different levels, with consistent expression identified at the mRNA and protein levels (Figure 3c,d,f), demonstrating that *THBS1* cell lines with stable knockdown and overexpression were successfully constructed.

### 2.4. Effect of Knockdown of THBS1 on Proliferation, Apoptosis, and Migration Ability of CL23 Cells

**Figure 4:**
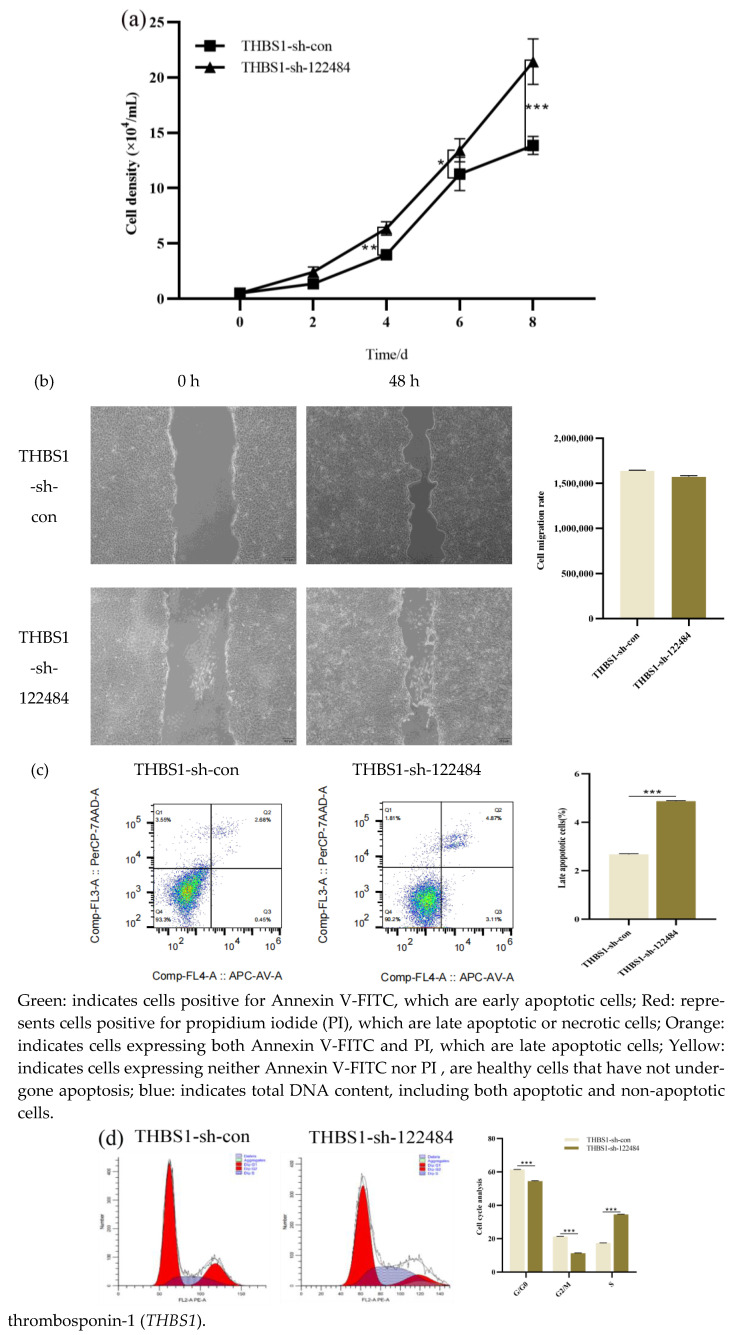
Effects of knockdown of *THBS1* on proliferation, apoptosis, migration, and cell cycle of CL23 cells. (**a**) Growth curves of stably knocked down *THBS1* cell lines. (**b**) Analysis of migratory ability of stably knocked down *THBS1* cell lines. (**c**) Analysis of apoptotic ability of stably knocked down *THBS1* cell lines. (**d**) Analysis of cell cycle of stably knocked down *THBS1* cell lines. * denotes statistically significant difference (* *p* < 0.05; ** *p* < 0.01; *** *p* < 0.001) and no * indicates no difference.

The proliferation rate (*p* < 0.05) (Figure 4a) and apoptosis level (*p* < 0.001) (Figure 4c) were significantly up-regulated on days 4–8 after the knockdown of *THBS1* compared with the knockdown control cells, whereas the difference in the cell migration abilities of the cell types was not significant (*p* > 0.05) (Figure 4b). The S-phase, which is the cellular DNA replication phase, was significantly up-regulated when *THBS1* was knocked down compared with the knockdown control group (*p* < 0.001) (Figure 4d), demonstrating that *THBS1* knockdown promoted the proliferative activity of CL23 cells.

### 2.5. Effects of Overexpression of THBS1 on Cell Proliferation, Apoptosis, and Migration Ability

**Figure 5:**
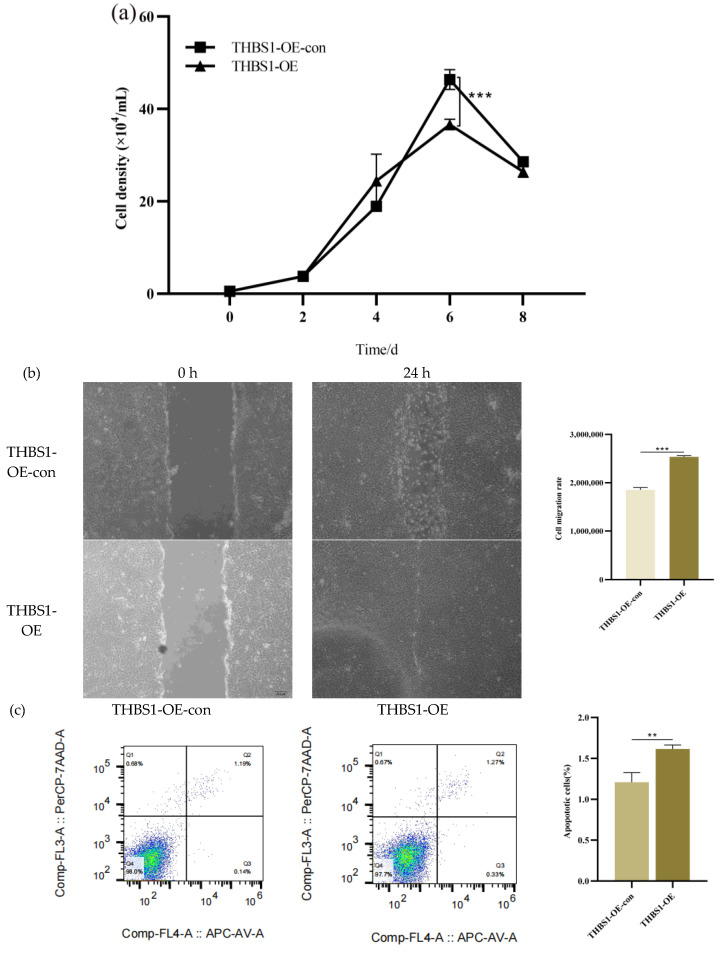

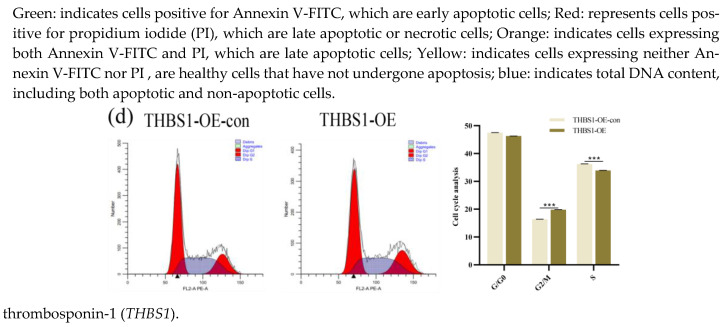
Effects of overexpression of *THBS1* on proliferation, apoptosis, migration, and cell cycle of M60 cells. (**a**) Growth curves of stable overexpression of *THBS1* cell lines. (**b**) Analysis of migration ability of stable overexpression of *THBS1* cell lines. (**c**) Analysis of apoptosis ability of stable overexpression of *THBS1* cell lines. (**d**) Analysis of cell cycle of stable overexpression of *THBS1* cell lines. * denotes statistically significant difference (** *p* < 0.01; *** *p* < 0.001) and no * indicates no difference.

Compared with the overexpression control cells, the proliferation rate was significantly reduced (*p* < 0.001) (Figure 5a), the apoptosis level was significantly up-regulated (*p* < 0.01) (Figure 5c), and the cell migration ability was significantly enhanced (*p* < 0.001) (Figure 5b) after the overexpression of *THBS1* on days 4–8. Moreover, the S-phase was significantly down-regulated (*p* < 0.001) (Figure 5d), further proving that *THBS1* overexpression inhibited the proliferative activity of M60 cells.

### 2.6. Effects of the Knockdown and Overexpression of THBS1 on H1N1 Influenza Virus Replication

**Figure 6:**
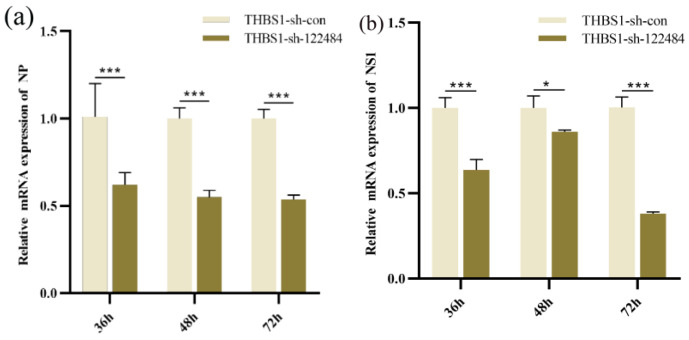

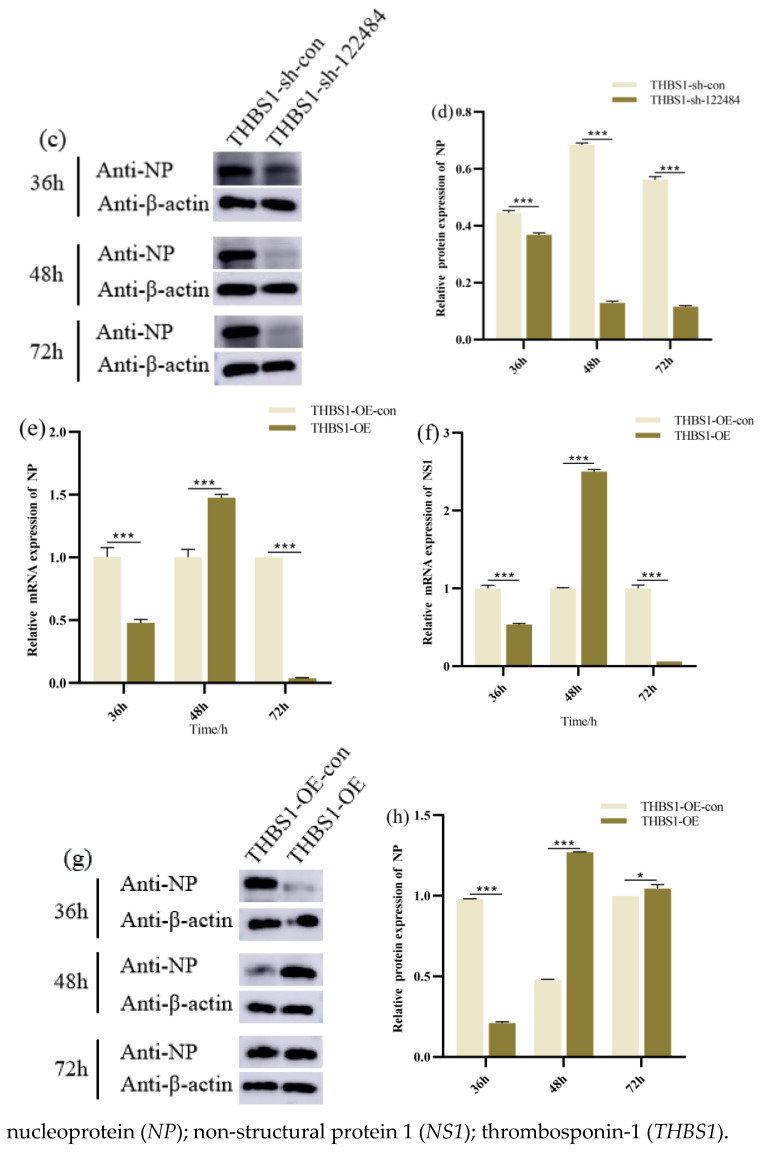
Effect of knockdown and overexpression of *THBS1* on H1N1 influenza virus replication in MDCK cells. (**a**,**b**) Differences in expression of *NP* and *NS1* mRNA levels across time of H1N1 influenza virus infection in stably knocked down *THBS1* cell lines. (**c**,**d**) Differences in expression of *NP* protein levels across time of H1N1 influenza virus infection in stably knocked down *THBS1* cell lines. (**e**,**f**) Stable overexpression of *THBS1* cell lines infected with H1N1 influenza virus different time period *NP* and *NS1* mRNA level expression differences. (**g**,**h**) Stable overexpression of *THBS1* cell lines infected with H1N1 influenza virus different time period NP protein level expression differences. * indicates statistically significant difference (* *p* < 0.05; *** *p* < 0.001) and no * indicates no difference.

*THBS1* knockdown significantly inhibited *NP* and *NS1* expression at the mRNA level (*p* < 0.05) (*p* < 0.001) (Figure 6a,b), and it also significantly inhibited *NP* expression at the protein level (*p* < 0.001) (Figure 6c,d) at 36 h, 48 h, and 72 h. THBS1 overexpression significantly promoted *NP* and *NS1* expression at the mRNA level at 48 h (*p* < 0.001) (Figure 6e,f), while significantly promoting *NP* expression at the protein level at 48 h and 72 h (*p* < 0.001) (*p* < 0.05) (Figure 6g,h). This indicates that *THBS1* knockdown inhibited H1N1 influenza virus replication in CL23 cells and *THBS1* overexpression promoted H1N1 influenza virus replication in M60 cells.

### 2.7. Expression of TGF-β/Smad Pathway Target Genes in M60 and CL23 Cells

**Figure 7:**
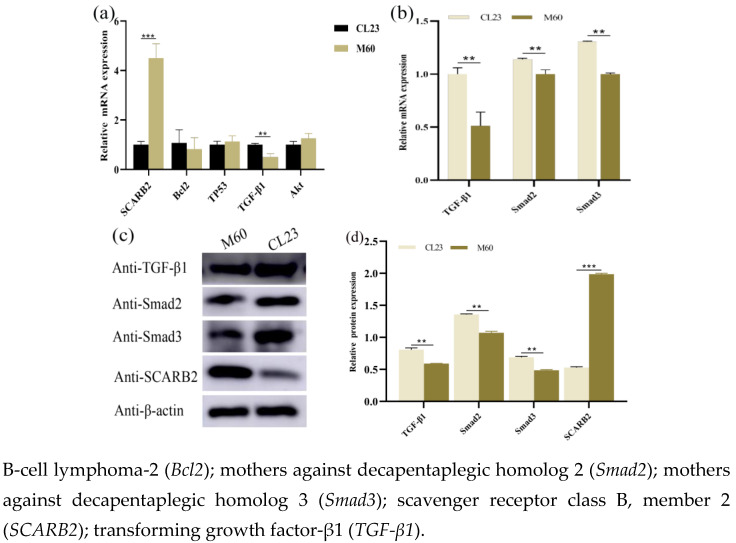
Differential expression of target genes downstream of *PI3K/Akt*, *P53*, and *TGF-β/Smad* signalling pathways, as well as the predicted THBS1-interacting gene, *SCARB2*, in M60 and CL23 cells. (**a**,**b**) Differential expression of target genes downstream of the *PI3K/Akt*, *P53*, and *TGF-β/Smad* signalling pathways, as well as *SCARB2* at mRNA level. (**c**,**d**) Differential expression of target genes downstream of the *TGF-β/Smad* signalling pathway and *SCARB2* at the protein level. * indicates statistically significant difference (** *p* < 0.01; *** *p* < 0.001) and no * indicates no difference.

Compared with in CL23 cells, the TGF-β/Smad signalling pathway downstream target genes *TGF-β1*, *Smad2*, and *Smad3* were significantly down-regulated at the mRNA and protein levels in M60 cells (*p* < 0.01), while *SCARB2* was significantly up-regulated (*p* < 0.001), and the *PI3K/Akt* and P53 signalling pathway downstream target genes *Akt*, *Bcl2*, and *TP53* were not significantly different at the mRNA level (*p* > 0.05) (Figure 7). We speculated that *THBS1* might be involved in MDCK cell biology regulation through *TGF-β/Smad* signalling, and that there might be a mutual inhibitory relationship between *SCARB2* and *THBS1*.

### 2.8. Effects of Knockdown and Overexpression of THBS1 on the Expression of Target Genes Downstream of TGF-β/Smad Signalling

**Figure 8:**
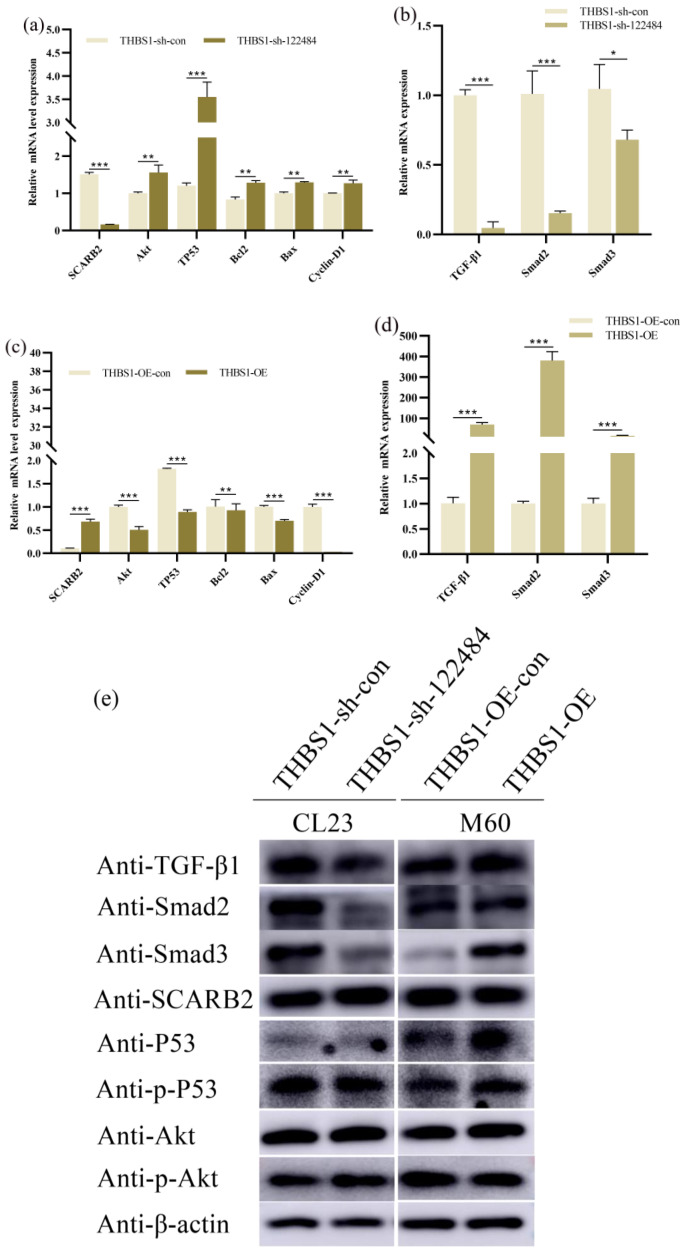

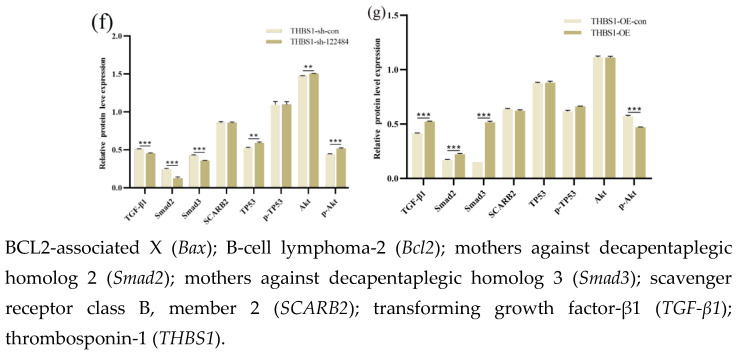
Effects of knockdown and overexpression of *THBS1* on the differential expression of target genes downstream of *TGF-β/Smad*, *PI3K/Akt*, *P53* signalling pathways, and *SCARB2* in MDCK cells. (**a**,**b**) Effects of knockdown of *THBS1* on the differential expression of target genes downstream of *PI3K/Akt*, *P53*, *TGF-β/Smad* signalling, and the *SCARB2* mRNA level expression differences in CL23 cells. (**c**,**d**) Differential effects of overexpression of *THBS1* on the expression of target genes downstream of *PI3K/Akt*, *P53*, *TGF-β/Smad* signalling, and *SCARB2* at the mRNA level in M60 cells. (**e**) Differential effects of knockdown and overexpression of *THBS1* on the expression of target genes downstream of the *PI3K/Akt*, *P53*, *TGF-β/Smad* signalling pathways in MDCK cells, and the *SCARB2* protein level expression differences. (**f**,**g**) Grey value analysis of knockdown and overexpression of *THBS1* on the expression of target genes downstream of the *PI3K/Akt*, *P53*, and *TGF-β/Smad* signalling pathways, as well as *SCARB2* protein level in MDCK cells. * indicates statistically significant difference (* *p* < 0.05; ** *p* < 0.01; *** *p* < 0.001) and no * indicates no difference.

To further determine the *THBS1* signalling pathways involved in proliferation and apoptosis regulation in MDCK cells and whether there was any interaction between *THBS1* and *SCARB2*, we performed differential expression assays of the downstream target genes of the *PI3K/Akt*, *P53*, and *TGF-/Smad* signalling pathways, as well as the predicted interacting gene *SCARB2*, with the THBS1-knockdown and -overexpression groups of cells. The results showed that, compared with knockdown control cells (*THBS1*-sh-con), the *TGF-β/Smad* signalling pathway downstream target genes of *TGF-β1*, *Smad2*, and *Smad3* were significantly down-regulated at both the mRNA and protein levels after THBS1 knockdown (*THBS1*-sh-122484) (Figure 8b,e,f). The *PI3K/Akt* pathway downstream target gene *Akt* and the *P53* pathway downstream target genes *TP53*, *Bcl2*, and *Bax* were significantly up-regulated at the mRNA level, while *SCARB2* and *Cyclin-D1* were significantly down-regulated at the mRNA level (Figure 8a), promoting phosphorylation at the *P53* protein level and inhibiting phosphorylation at the *Akt* protein level. Moreover, *SCARB2* was significantly non-significant at the protein level (Figure 8e,f). This indicates that the knockdown of *THBS1* in CL23 cells could inhibit *TGF-β/Smad* signalling, activate *P53* phosphorylation, and inhibit *Akt* phosphorylation in CL23 cells. Furthermore, *THBS1* overexpression in M60 cells could activate *TGF-β/Smad* signalling, inhibit *P53* phosphorylation, and promote *Akt* phosphorylation in M60 cells. Therefore, we speculated that *THBS1* regulated the proliferation and apoptosis of MDCK cells through *TGF-β/Smad* signalling, whereas after *THBS1* knockdown and overexpression, *SCARB2* only underwent changes at the mRNA level, not the protein level, which might be related to the post-transcriptional translation in the cells. Therefore, we speculated that there was no interaction between the *THBS1* and *SCARB2* proteins.

### 2.9. Effects of TGF-β Activators and Inhibitors on TGF-β/Smad Signalling Target Genes and THBS1 Expression in MDCK Cells

**Figure 9:**
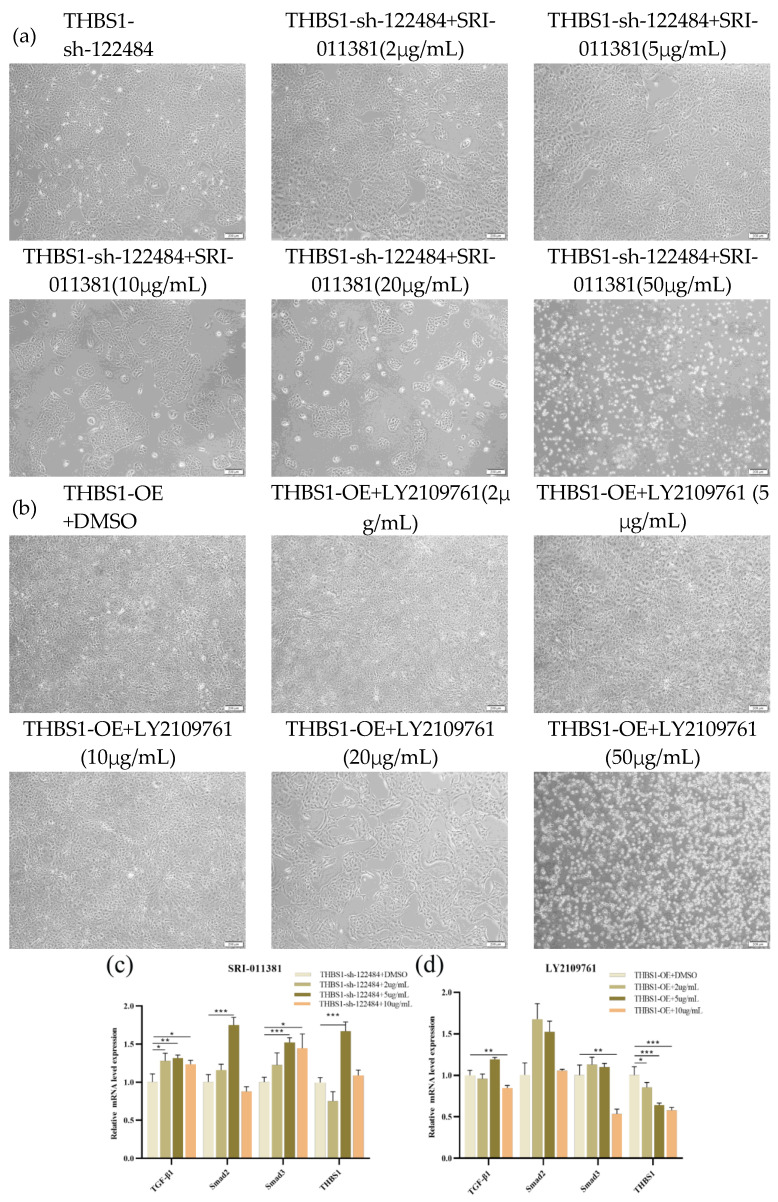

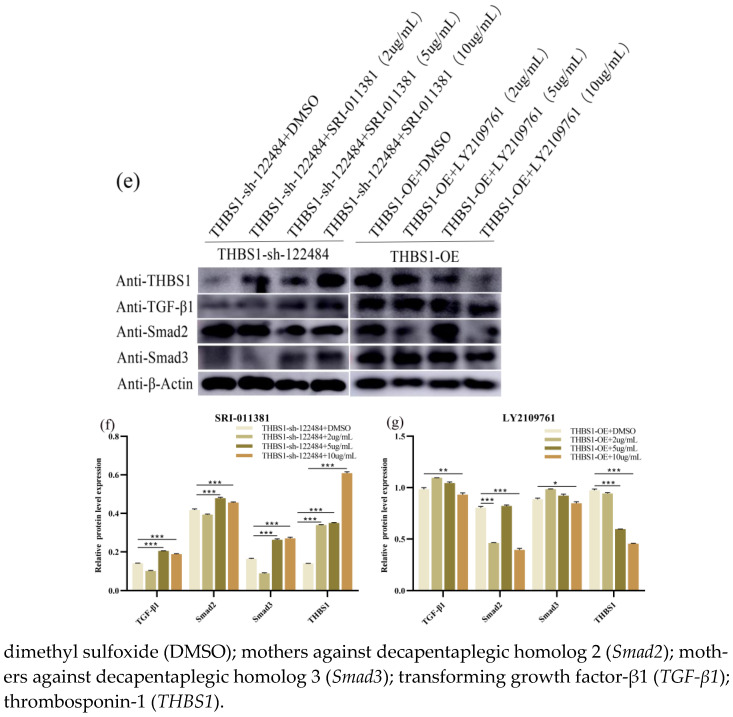
Determination of optimal concentration of *TGF-β* activator SRI-011381 on *THBS1*-sh-122484 cells and inhibitor LY2109761 on *THBS1*-OE cells, and its effect on the expression of target genes, *THBS1*, downstream of *TGF-β/Smad* signalling. (**a**) Effect of different concentrations of SRI-011381 on the growth status of *THBS1*-sh-122484 cells. (**b**) Effects of different concentrations of LY2109761 on the growth status of *THBS1*-OE cells. (**c**) Effects of different concentrations of SRI-011381 on the differential expression of target genes downstream of *TGF-β/Smad* signalling and *THBS1* mRNA levels in *THBS1*-sh-122484 cells. (**d**) Effects of different concentrations of LY2109761 on the expression of target genes downstream of *TGF-β/Smad* signalling and *THBS1*, and effects of different concentrations of LY2109761 on the expression of target genes downstream of *THBS1*-Sh-122484 cells. LY2109761 on *THBS1*-OE cells *TGF-β/Smad* signalling downstream target genes and *THBS1* mRNA level expression differences. (**e**) Effect of different concentrations of SRI-011381 on *THBS1*-sh-122484 cells and different concentrations of LY2109761 on *THBS1*-OE cells *TGF-β/Smad* signalling downstream target genes and *THBS1* protein level expression differences. (**f**) Grey scale analysis of the effects of different concentrations of SRI-011381 on the expression of target genes and *THBS1* protein level downstream of *TGF-β/Smad* signalling in *THBS1*-sh-122484 cells. (**g**) The effects of different concentrations of LY2109761 on the expression of *TGF-β/Smad* signalling downstream of *TGF-β/Smad* protein in *THBS1*-OE cells target gene and *THBS1* protein level expression grey value analysis. * indicates statistically significant difference (* *p* < 0.05; ** *p* < 0.01; *** *p* < 0.001) and no * indicates no difference.

The results showed that in the positive control group (*THBS1*-sh-122484+DMSO) with DMSO, the growth state of adherent cells gradually decreased from the 10 μg/mL concentration, and more than 90% of the cells were in the floating state at the 50 μg/mL concentration (Figure 9a). The detection of differences in the expression of downstream target genes of the *TGF-β/Smad* signalling pathway and *THBS1* in cells in the 2 μg/mL, 5 μg/mL, and 10 μg/mL concentration groups, as well as in the positive control group, revealed that *TGF-β1*, *Smad2*, *Smad3*, and *THBS1* activation at the mRNA and protein levels was significant at 5 μg/mL compared with that of the positive control group (Figure 9c,e,f). In the DMSO positive control group (*THBS1*-OE+DMSO), the growth status of adherent cells gradually decreased from 20 μg/mL, and more than 90% of cells were floating at a 50 μg/mL concentration (Figure 9b). Detecting differences in the expression of downstream target genes of the *TGF-β/Smad* signalling pathway and *THBS1* in cells in the 2 μg/mL, 5 μg/mL, and 10 μg/mL concentration groups, as well as in the positive control group, revealed that the inhibitory effects of *TGF-β1*, *Smad3*, and *THBS1* at the mRNA and protein levels were significant at 10 μg/mL compared with those in the positive control group (Figure 9d,e,g). This indicated that the optimal activation concentration of SRI-011381 for *THBS1*-knockdown cells (*THBS1*-sh122484) was 5 μg/mL, while the optimal inhibitory concentration of LY2109761 for *THBS1*-overexpressing cells (*THBS1*-OE) was 10 μg/mL. Meanwhile, we also confirmed that *THBS1* regulated MDCK cells by inhibiting the *TGF-β/Smad* signalling pathway to regulate MDCK cell proliferation and apoptosis.

### 2.10. Effects of TGF-β Activators and Inhibitors on Target Genes Downstream of PI3K/Akt and P53 Signalling in MDCK Cells

**Figure 10:**
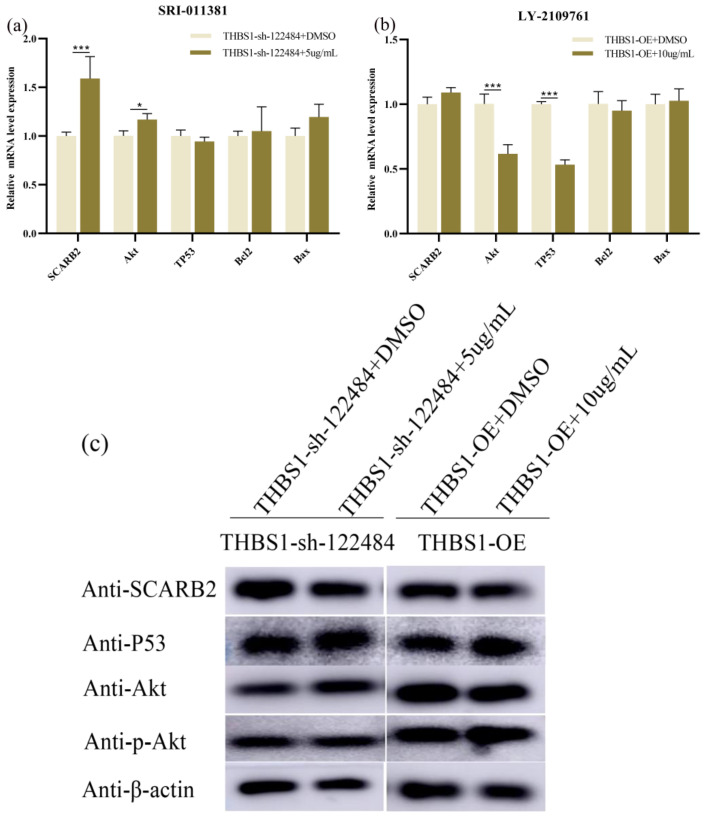

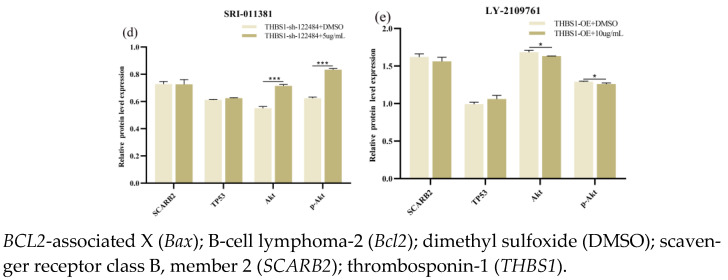
Effect of SRI-011381 (5 μg/mL) on the expression of *PI3K/Akt*, target genes downstream of *P53* signalling pathway, *SCARB2* in knockdown *THBS1* cells (*THBS1*-sh-122484), and LY2109761 (10 μg/mL) in overexpressing *THBS1* (*THBS1*-OE) cells. (**a**) SRI-011381 (5 μg/mL) intervention in *THBS1*-sh-122484 cells showed differential expression of *PI3K/Akt*, target genes downstream of *P53* signalling, and *SCARB2* at the mRNA level. (**b**) LY2109761 (10 μg/mL) intervention in *THBS1*-OE cells showed differential expression of *PI3K/Akt*, target genes downstream of *P53* signalling, and *SCARB2* expression differences at the mRNA level. (**c**) *PI3K/Akt*, *P53* signalling downstream target genes, and *SCARB2* expression differences at the protein level after SRI-011381 (5 μg/mL) intervention in *THBS1*-sh-122484 cells and LY2109761 (10 μg/mL) intervention in *THBS1*-OE cells. (**d**) SRI-011381 (5 μg/mL) intervention in *THBS1*-sh-122484 cells after *PI3K/Akt*, *P53* signalling downstream target genes, and *SCARB2* expression differences at protein level grey value analysis. (**e**) LY2109761 (10 μg/mL) intervention in *THBS1*-OE cells after *PI3K/Akt*, *P53* signalling downstream target genes, and *SCARB2* expression difference at protein level grey value analysis. * indicates statistically significant difference (* *p* < 0.05; *** *p* < 0.001) and no * indicates no difference.

We investigated the effects of *TGF-β* activators and inhibitors on the knockdown and overexpression of *PI3K/Akt* and *P53* downstream target genes and *SCARB2* in *THBS1* cells. *Akt* and *SCARB2* were significantly up-regulated at the mRNA level, while *TP53*, *Bcl2*, and *Bax* were not significantly changed at the mRNA level in *THBS1*-sh-122484 cells after SRI-011381 (5 μg/mL) intervention, compared to the DMSO positive control group (Figure 10a). The phosphorylation of the *Akt* and *SCARB2* proteins was not significant (Figure 10c,d), *Akt* and *TP53* were significantly up-regulated at the mRNA level, while *SCARB2*, *Bcl2*, and *Bax* were not significantly modulated at the mRNA level in *THBS1*-OE cells after LY2109761 intervention (10 μg/mL) (Figure 10b). The phosphorylation of the *Akt* protein was inhibited, and the difference in the *SCARB2* protein level was not significant (Figure 10c,e). This indicates that SRI-011381 could activate *Akt* phosphorylation in *THBS1*-knockdown cells (*THBS1*-sh122484), and LY2109761 could inhibit such phosphorylation in *THBS1*-overexpressing cells (*THBS1*-OE), in turn regulating MDCK proliferation and apoptosis.

### 2.11. Effects of TGF-β Activators and Inhibitors on the Proliferative, Migratory Capacities and Apoptosis Ability of THBS1-Knockdown and -Overexpressing Cells

**Figure 11:**
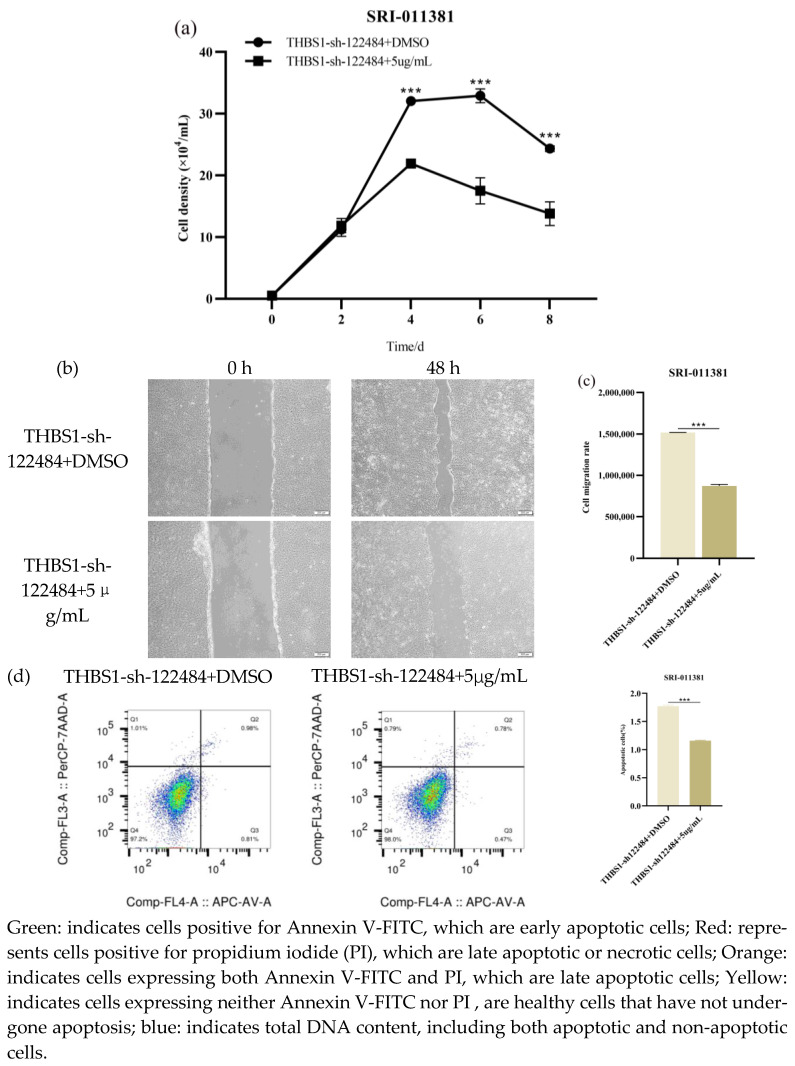

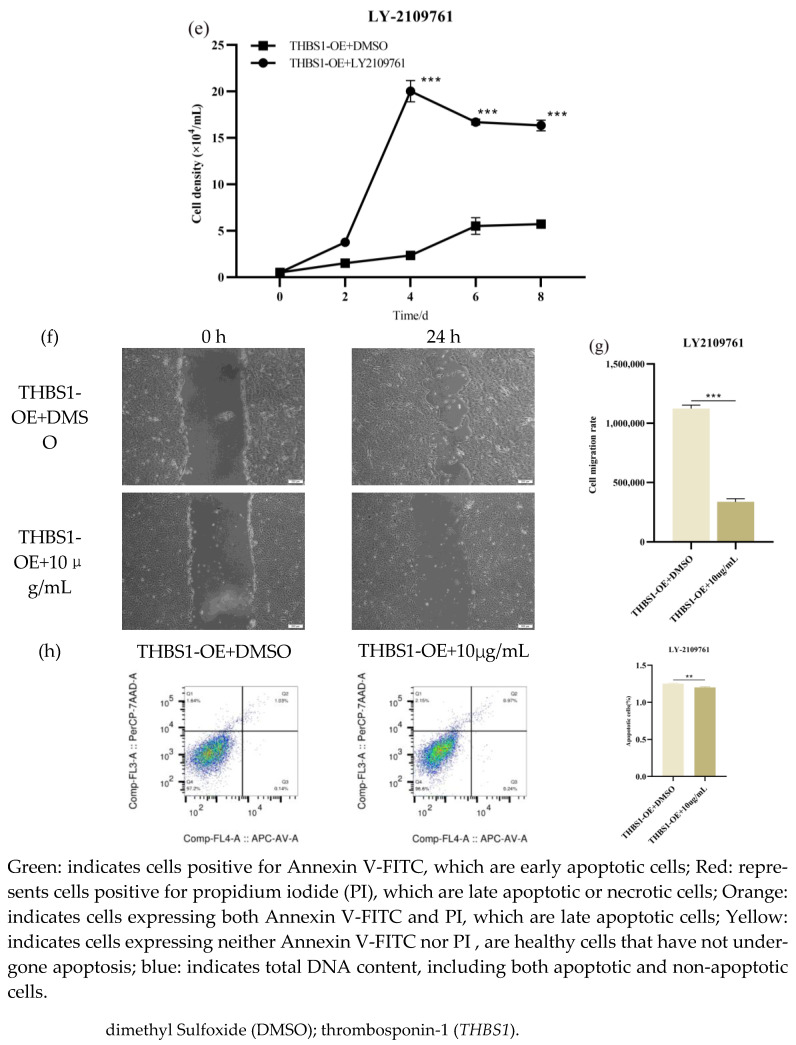
Effects of SRI-011381 on knockdown *THBS1* cells (*THBS1*-sh-122484) and LY2109761 on proliferation and migration ability of overexpressing *THBS1* cells (*THBS1*-OE). (**a**) Growth curve of SRI-011381 intervention knockdown *THBS1* cells (*THBS1*-sh-122484). (**b**,**c**) SRI-011381 intervention knockdown *THBS1* cells (*THBS1*-sh-122484) migration ability assay. (**d**) SRI-011381 intervention knockdown *THBS1* cells (*THBS1*-sh-122484) apoptosis ability assay. (**e**) LY2109761 intervention overexpression *THBS1* cells (THBS1-OE) growth curve. (**f**,**g**) LY2109761 intervention overexpression *THBS1* cells (*THBS1*-OE) migration ability assay. (**h**) LY2109761 intervention overexpression *THBS1* cells (THBS1-OE) apoptosis ability assay. * indicates statistically significant difference (** *p* < 0.01; *** *p* < 0.001) and no * indicates no difference.

In this study, we evaluated the effects of SRI-011381, a signalling pathway activator, on the proliferative, migratory capacity and apoptosis ability of *THBS1*-knockdown cells (*THBS1*-sh122484) and the effects of the inhibitor LY2109761 on the proliferative, migratory capacity and apoptosis ability of *THBS1*-overexpressing cells (*THBS1*-OE) to further determine *THBS1*’s influence on the proliferative and apoptosis capacity of MDCK cells, by regulating *TGF-β* signalling capacity. Compared with that in the DMSO positive control group, the proliferation rate of *THBS1*-knockdown cells (*THBS1*-sh122484) in the SRI-011381-treated group was significantly down-regulated after day 2 (*p* < 0.001) (Figure 11a), the cell migration rate was significantly reduced at 48 h (*p* < 0.001) (Figure 11b,c), and the cell apoptosis ability was significantly reduced (*p* < 0.001) (Figure 11d). The proliferation rate of *THBS1*-overexpressing cells (*THBS1*-OE) in the LY2109761 treatment group was significantly up-regulated after day 2 (*p* < 0.001) (Figure 11e), the cell migration rate was significantly reduced at 24 h (*p* < 0.001) (Figure 11f,g), and the cell apoptosis ability was significantly reduced (*p* < 0.01) (Figure 11h). This indicates that SRI-011381 reduced the proliferation, migration, and apoptosis ability of *THBS1*-knockdown cells (*THBS1*-sh122484. Moreover, LY2109761 promoted the proliferation of *THBS1*-overexpressing cells (*THBS1*-OE) and inhibited their migration and apoptosis ability.

## 3. Discussion

MDCK cells are considered the most favourable cellular substrate for influenza vaccine production, due to their specific efficacy in becoming infected with influenza viruses, rapid proliferation, and reduced susceptibility to mutations [28]. However, the tumourigenic phenotype possessed by MDCK cells is highly controversial during vaccine production [29], and in recent years, much evidence has highlighted key factors of MDCK cell tumourigenicity, such as *CDC20*, *TGM2*, and *miR-2779-x*. Moreover, a previous study demonstrated that they regulate influenza virus replication [30,31], as well as affecting the proliferative capacity of MDCK cells, by influencing the oncogenic phenotype of MDCK cells. *IncRNAMSTRG.1056.2* has also been shown to regulate *ERBB3*, which activates the *PI3K-Akt* pathway and promotes tumourigenic phenotype development in MDCK cells [32].

MDCK cells greatly support the proliferation of most IVAs; therefore, they are widely used in influenza vaccine production. Increasing the susceptibility of MDCK cells to influenza viruses can increase the vaccine yield or lead to the isolation of more strains of influenza viruses, which is important for controlling influenza virus transmission [5]. Many studies on influenza virus replication in MDCK cells and virus-associated regulatory mechanisms have been reported in recent years. CRISPR-Cas9 gene-editing technology was used to construct *TRAF3*-knockout MDCK cells (MDCK-*TRAF3*^-/-^), and after IVA infection, the HA titre and viral titre of MDCK-*TRAF3*^-/-^ cells were elevated, the type I interferon-related pathway was significantly blocked, and the transcription of several antiviral-related genes was significantly reduced; therefore, the knockout of the *TRAF3* gene decreased the resistance of MDCK cells to IVA, thus providing a prospective research field for improving IVA isolation and influenza vaccine production [33]. After successive passages in cell culture, influenza viruses may undergo HA and NA protein mutations, reducing the effectiveness of the vaccine [34]. Therefore, researchers compared the HA and NA sequences obtained from the microRNA inhibitor-treated group with the parental population used and found that neither HA nor NA were mutated, despite virus propagation in cells treated with microRNA inhibitors and gene-editing tools that knock out or knock down microRNAs both in vitro and in vivo [35,36,37].

In this study, we found, for the first time, that interfering with *THBS1* affected IVA replication in MDCK cells, and studies on the effect of *THBS1* on influenza virus replication have not yet been reported, so *THBS1* may be a potential target gene affecting influenza virus replication, showing potential promise for the study of potential influenza vaccines for the matrix of MDCK cells. Regarding the regulation of IVA replication by THBS1, the replication mechanism in MDCK cells needs to be further explored.

*THBS1*, a major component of human platelets, is an extracellular matrix glycoprotein synthesised and secreted by various cells, including platelets, fibroblasts, and vascular endothelial cells [38,39]. *THBS1* binds to extracellular matrix ligands, including fibrinogen, fibronectin, some collagens, latent and active *TGF-β1*, *TNFAIP6* (*TSG6*), heparin, fibronectin, *CTSG* (histone G), *ELANE* (neutrophil elastase), some *MMPs*, tissue-factor pathway inhibitors, and heparan sulphate proteoglycans, and it participates in signalling pathways that regulate cell proliferation and apoptosis via *CALR* and integrin binding to the cell surface receptors *CD36*, *CD47*, and *LRP1* [40,41]. In this study, we found that *THBS1* was highly expressed in CL23 cells, and their proliferation, apoptosis, and migration abilities were lower than those of M60 cells, so we hypothesised that *THBS1*’s ability to participate in proliferation and apoptosis regulation in MDCK cells was related to cellular tumourigenicity. To confirm this theory, we interfered with the expression of *THBS1* in MDCK cells with different tumourigenicities in vitro and demonstrated that THBS1 regulated MDCK cell proliferation, apoptosis, migration and cell cycles, suggesting that THBS1 influences MDCK cell tumourigenicity by affecting MDCK cell proliferation and apoptosis capacity, as well as the cell cycle. It has been shown that *THBS1* stimulates the migration and proliferation of vascular endothelial cells and capillary bud formation in vitro [42]. Jiang, D. et al. [11] found that *THBS1* overexpression significantly induced the proliferation and migration of proliferative scar fibroblasts while inhibiting apoptosis, and *THBS1* silencing mildly suppressed fibroblast proliferation and migration and up-regulated apoptosis. *THBS1* also promoted proliferation and inhibited apoptosis in RAW264.7 cells and promoted tumour cell proliferation, migration, growth, and survival in vivo [43,44].

*TGF-β* is a key cytokine that regulates T-cell development, activation, proliferation, differentiation, and death [45]. The *TGF-β* superfamily is known to have at least 33 members, including *TGF-β*, bone morphogenetic proteins (BMPs), activins, inhibins, and glial cell lineage-derived neurotrophic factors (*GDNFs*) [46]. *TGF-β1* is the predominant isoform found in blood and immune cells, and it plays a key role in regulating the immune response [47]. *TGF-β* regulates various biological processes such as tissue homeostasis, angiogenesis, migration, and differentiation [48]. *TGF-β* stimulation stops proliferation in almost all non-carcinogenic epithelial cells, endothelial cells, haematopoietic cells, and neuronal cells, and some mesenchymal cells [49]. During haematopoiesis, the *TGF-β* signalling pathway is a key negative regulator of proliferation and promotes differentiation and apoptosis under appropriate circumstances. In certain haematological malignancies, *TGF-β* also has tumour suppressor activity [50].

In this study, we found that *THBS1* knockdown promoted proliferation and apoptosis in CL23 cells and inhibited the expression of *TGF-β1* and *Smad2/3*, as well as inhibiting *Akt* phosphorylation and promoting *P53* phosphorylation, whereas the opposite result was observed in M60 cells, which suggests that *THBS1* can regulate the proliferation and apoptosis ability of DMCK cells through *TGF-β/Smad* signalling, and may also be involved in the *PI3K/Akt/P53* axis that regulates the oncogenic capacity of MDCK cells. *TGF-β* plays a balancing role by inducing cell cycle arrest and apoptosis, and in some haematological malignancies, it also has tumour suppressor activity. The *TGF-β/Smad* signalling pathway can cause growth inhibition, differentiation, and apoptosis [51,52,53]. To further validate this possible regulatory process, we pharmacologically intervened in MDCK cell lines with *THBS1* knockdown and overexpression using *TGF-β* activators and inhibitors, finding that *TGF-β* activators and inhibitors reversed the effects on the expression and *Akt* phosphorylation of *THBS1*, *TGF-β1*, and *Smad2/3*, as well as reversing the effect on cellular proliferative capacity, demonstrating that *THBS1* is regulated through *TGF-β/Smad* signalling, which regulates the proliferative and apoptotic capacity of DMCK cells and cellular tumourigenicity development. *TGF-β* activates various intracellular signalling pathways, including the classic *Smad* pathway. Upon activating *TGF-β* signalling, *TGF-β* transmits signals by binding to specific heterodimeric type I and II receptor complexes. *TGF-β* first binds to *Tβ RII*, and it then recruits and activates *Tβ RI*. The activated *Tβ RI* then recruits and phosphorylates the receptor *Smad*, *Smad2/3* induces *TGF-β* phosphorylation at the C-terminus or linker region, and phosphorylated *Smad2/3* forms a complex with *Smad4* for translocation to the nucleus to regulate its target genes [54,55]. In general, *Smad2/3/4* complexes cannot regulate the transcription of target genes without interacting with other co-transcription factors, and *Smad3* and *Smad2* compensate for each other by mediating *TGF-β* signalling in immune cells, as the absence of *Smad2* and *Smad3* in T cells completely blocks *TGF-β* signalling [56]. In addition, Soo Min Lee et al. [57] found that *THBS1* is involved in both the *TGF-β* and *p53* pathways, and they demonstrated through in vitro experiments that the main mechanism is the regulation of a positive feedback loop between the *THBS1* and *TGF-β* pathways.

Past studies show that MDCK cells are an ideal cellular matrix for influenza vaccine production compared to other available cellular matrices. Increasing influenza virus titres and reducing tumour formation in MDCK cells are issues that must be addressed [4]; however, our findings indicate that *THBS1* plays a role in regulating MDCK cell proliferation and apoptosis through *TGF-β/Smad* signalling. Additionally, preliminary evidence suggests that *THBS1* may influence the expression of the H1N1 influenza virus nuclear gene *NP*. Nevertheless, the precise impact of *THBS1* on influenza virus titer and its underlying mechanism remains unclear. It is our intention, therefore, to enhance influenza virus replication and titer by identifying MDCK cell proliferation-related targets to increase cell proliferation efficiency. Furthermore, we will investigate the regulatory mechanism of *THBS1* on influenza virus replication and its effect on the enhancement of influenza virus titer in future studies.

In conclusion, in this study, we found for the first time that *THBS1* regulated MDCK cell proliferation and apoptosis through the *TGF-β/Smad* signalling pathway and, at the same time, participated in *PI3K/Akt/P53* axis signalling regulation, which may be related to the *THBS1* feedback regulation mechanism of the *TGF-β* pathway. We also found that *THBS1* regulated influenza virus replication. We hope to further understand the role of *THBS1* in MDCK tumourigenicity and influenza virus replication, and to better understand the function of *THBS1* in MDCK cells affecting influenza virus replication, providing a basis for the construction of high-yield genetically engineered MDCK cell lines for vaccine use.

## 4. Material and Methods

### 4.1. Cell Culture and Virus

MDCK-M60 cells were purchased from American Type Culture Collection (ATCC) and provided by the Gansu Animal Cell Technology Innovation Centre; non-tumourigenic MDCK-CL23 cells were domesticated and cultured by the Gansu Animal Cell Technology Innovation Centre. Frozen MDCK-M60 and MDCK-CL23 cells were resuscitated in T25 square flasks. They were mixed with 5 mL of 10% NBS/DMEM medium or 5 mL of 10% FBS/DMEM medium (Minhai Bioengineering, Lanzhou, China), respectively, and cultured in a 37 °C, 5% CO_2_ incubator, and the medium was changed after 12 h to 24 h. The H1N1 influenza virus strain number was X-275 and was provided by the Wuhan Institute of Biological Products Co., Ltd. (Wuhan, China).

### 4.2. Lentivirus Transfection

The *THBS1* knockdown and overexpression lentiviruses used in this experiment were synthesised by Gikai. MDCK cells (M60, CL23 cells) with good growth statuses were detached with the help of trypsin and counted, before being inoculated into 24-well plates at 5 × 10^4^ cells/well, and cells were grown in an incubator with 5% CO_2_ at a temperature of 37 °C. When the cells had grown to 60–70% confluence, they were detached with the help of trypsin and counted. Then, the volume of the viral fluid required per well was calculated according to the number of cells and the viral titre, and the volume of lentivirus in each well was calculated according to MOI = 100. *THBS1* RNAi (122484-1, 122485-2, and 122486-2) and a negative control CON313 lentiviral solution were inoculated into MDCK (CL23) cells, and LV-THBS1 (93180-22) and a negative control CON335 lentiviral solution were inoculated into MDCK (M60) cells in the overexpression group at MOI = 100. The cells were cultured for 12 h and the medium was then replaced with 10% NBS DMEM normal medium. The fluorescence expression intensity was observed under a fluorescence microscope after 24 h, and the cells were passaged, frozen, and tested for the cell transient effect. Lentivirus-transfected cells were passaged and cultured, and 4 μg/mL puromycin (Solarbio, Beijing, China) added to the medium was used for cell resistance screening when the cells had grown to 20–30% confluence. The fluorescence expression intensity was then observed under a fluorescence microscope over 2–4 generations under puromycin treatment; when the fluorescence expression effect reached 98% or more, the THBS1 expression level was verified. The successful construction of *THBS1*-knockdown and -overexpression cell lines was verified using the fluorescence expression intensity and *THBS1* expression levels of the cells, which were then prepared for detecting the effects of *THBS1* on their proliferation and apoptosis phenotypes.

### 4.3. Treatment of THBS1-Knockdown/Overexpression Cell Lines with TGF-β Activator and Inhibitor

To further evaluate the mechanism by which *THBS1* regulates MDCK cell proliferation and apoptosis through *TGF-β/Smad* signalling, we observed the cell growth status with different concentration gradients of the *TGF-β/Smad* signalling pathway activator SRI-011381 (MCE, Shanghai, China, HY-12075) of THBS1-knockdown cells (*THBS1*-sh122484) and the inhibitor LY2109761 (MCE, Shanghai, China, HY-100347) of *THBS1*-overexpressing cells (*THBS1*-OE). We also identified the optimal concentrations of SRI-011381 for *THBS1*-knockdown cells (*THBS1*-sh122484) and LY2109761 for *THBS1*-overexpressing cells (*THBS1*-OE) to further determine *THBS1*’s involvement in the regulation of the *TGF-β/Smad* signalling pathway.

In total, 5 mg each of the *TGF-β* pathway activator SRI-011381 and inhibitor LY2109761 powder was added to 5 mL of dimethyl Sulfoxide (DMSO) solution (Solarbio, Beijing, China) to make a master mix of 1 mg/mL, which was stored in an ultra-low-temperature freezer at −80 °C. *THBS1*-sh-122484 and THBS1-OE cells with good growth statuses were added to 6-well plates, and after they reached 80% confluence, they were washed three times with PBS, and treated with 8 μL, 20 μL, 40 μL, 80 μL, or 200 μL of activator or inhibitor; the control cells received the same volume of DMSO. The stock solutions were added to 4 mL of serum-free DMEM medium to form 2 μg/mL, 5 μg/mL, 10 μg/mL, 20 μg/mL, or 50 μg/mL concentrations, before being added to a 6-well plate with the cells and placed in a 37 °C, 5% CO_2_ incubator for 3 days. Then, the state of the cells in each treatment group was observed under a microscope and photographed, and at the same time, the effects of the concentration of each treatment (*THBS1*-sh-122484 + DMSO, *THBS1*-sh-122484 + SRI011381 (2 μg/mL), *THBS1*-sh-122484 + SRI011381 (5 μg/mL), *THBS1*-sh-122484 + SRI011381 (10 μg/mL), *THBS1*-OE + DMSO, *THBS1*-OE + LY2109761 (2 μg/mL), *THBS1*-OE + LY2109761 (5 μg/mL), and *THBS1*-OE + LY2109761 (10 μg/mL)) were determined. RNA and protein samples were analysed to detect downstream pathway target genes and the effects of *THBS1* activation and inhibition.

### 4.4. RNA Extraction and cDNA Library Construction

The following cells were cultured and prepared: MDCK-M60 and MDCK-CL23 *THBS1*-sh-con, *THBS1*-sh-122484, *THBS1*-sh-122485, *THBS1*-sh-122486, *THBS1*-OE-con, and *THBS1*-OE cells; *THBS1*-sh-122484 + DMSO, *THBS1*-sh-122484 + SRI011381 (2 μg/mL), *THBS1*-sh-122484 + SRI011381 (5 μg/mL), and *THBS1*-sh-122484 + SRI011381 (10 μg/mL) cells; and *THBS1*-OE + DMSO, *THBS1*-OE + LY2109761 (2 μg/mL), *THBS1*-OE + LY2109761 (5 μg/mL), and *THBS1*-OE + LY2109761 (10 μg/mL) cells. We extracted RNA using the TRIzol method following the manufacturer’s instructions, and used 1–2 μL of each RNA sample to detect the absorbance at 450 nm. For the detection of RNA extraction concentration and purity, a concentration range of 300–1500 μg/μL was used and an absorbance ratio of 1.8–2.0 was taken to indicate purity.

### 4.5. Reverse Transcription and RT-qPCR of RNA Samples

The Evo M-MLV Reverse Transcription Premix Kit was purchased from Acer Biologics. Total RNA was isolated and extracted using TRIzol reagent, and then cDNA was synthesised using the Evo M-MLV Reverse Transcription Premix Kit. cDNA from MDCK-M60 and MDCK-CL23 cells was used as the template for RT-qPCR. The expression level of the target gene was detected via PCR. The primer sequences are shown in Table 1. The reaction conditions were as follows: pre-denaturation at 95 °C for 30 s, denaturation at 95 °C for 10 s, and annealing at 60 °C for 30 s, with a total of 40 cycles. The denaturation curve was analysed at 65 °C for 5 s and 95 °C for 5 min. After the reaction was complete, the data were analysed using F = 2^−ΔΔΔCt^, and graphs were generated using GraphPad Prism 8 for differential expression analysis of the target genes.

### 4.6. Western Blotting

We separated the proteins by SDS-PAGE and then transferred them to a membrane. At the end of the experiment, the membrane was blocked with 5% skimmed milk powder in Tris-buffered saline with Tween 20 (TBST) solution for 2 h, and after blocking incubation with the antibody takes place straight away. The *THBS1* primary antibody (Abcam, Shanghai, China, ab267388; 1:1000 dilution), *EPHB2* primary antibody (Abcam, Shanghai, China, ab307811; 1:1000 dilution), *TGF-β1* primary antibody (Bioss, Beijing, China, bs-0086R; 1:1000 dilution), *Smad2* primary antibody (Bioss, Beijing, China, bs-0718R; 1:1000 dilution), *Smad3* primary antibody (Affinity Biologicals, Ancaster, ON, Canada, AF6362; 1:1000 dilution), *TP53* primary antibody (Abcam, Shanghai, China, ab26; 1:1000 dilution), *p-TP53* primary antibody (MCE, Shanghai, China, HY-P80472; 1:1000 dilution), *Akt* primary antibody (Affinity Biologicals, USA, AF0836; 1:1000 dilution), *p-Akt* primary antibody (Affinity Biologicals, USA, AF0832; 1:1000 dilution), *SCARB2* primary antibody (Abcam, Shanghai, China, ab26; 1:1000 dilution), and endogenous *β-actin* primary antibody (Abcam, Shanghai, China, ab8226; 1:2000 dilution) were incubated with the membrane at room temperature for 2 h or overnight at 4 °C. Then, the membrane was washed three times with TBST (5 min/times) and the goat anti-rabbit IgG secondary antibody (Bioss, Beijing, China, bs-80295G-HRP;1:5000 dilution) was incubated with it at room temperature for 1 h. It was then washed three times with TBST (5 min/time) and photographed after colour development via the ECL method. Then, the grey value of the protein bands was determined using Image J 1.8.0 software, and the protein grey expression was statistically analysed using GraphPad Prism 8.0 software.

### 4.7. Cell Proliferative Capacity Assay

Well-grown MDCK-M60, MDCK-CL23, *THBS1*-sh-con, *THBS1*-sh-122484, *THBS1*-OE-con, *THBS1*-OE, *THBS1*-sh-122484 + DMSO, *THBS1*-sh-122484 + SRI011381 (5 μg/mL), *THBS1*-OE + DMSO, and *THBS1*-OE + LY2109761 (10 μg/mL) cells were detached with the help of trypsin and counted, 24-well plates were seeded at 0.5 × 10^4^ cells/mL, and 1 mL of 10% NBS DMEM medium was added to each well. Then, counting was carried out for 8 consecutive days, and the average value was calculated from the three replicate wells of each group to plot the cell growth curve.

### 4.8. Detection of Apoptotic Capacity

MDCK-M60, MDCK-CL23, *THBS1*-sh-con, *THBS1*-sh-122484, *THBS1*-OE-con, and *THBS1*-OE cells with good growth statuses were taken from T25 cell vials. A single-cell suspension was prepared and stained with Annexin/PI and analysed by flow cytometry. FITC has green fluorescence, PI has red fluorescence, and the apoptosis level of the cells was analysed.

### 4.9. Cell Migration Assays

Well-grown MDCK-M60, MDCK-CL23, *THBS1*-sh-con, *THBS1*-sh-122484, *THBS1*-OE-con, *THBS1*-OE, *THBS1*-sh-122484 + DMSO, *THBS1*-sh-122484 + SRI011381 (5 μg/mL), *THBS1*-OE + DMSO, and *THBS1*-OE + LY2109761 (10 μg/mL) cells were detached with the help of trypsin and spread on 6-well plates. Then, 3 mL of 10% NBS DMEM medium was added to each well, and when the cells reached 100% confluence, they were scratched with a pipette tip. The data of the cell healing degree were analysed using Image J software, and difference analysis was performed using GraphPad Prism 8.0 statistical software.

### 4.10. Analysing the Cell Cycle Viaflow Cytometry

We used *THBS1*-sh-con, *THBS1*-sh-122484, *THBS1*-OE-con, and *THBS1*-OE cells with good growth statuses after they had reached confluence in T25 cell bottles, discarded the medium, washed them with PBS three times, added 1 mL of pancreatic enzyme to detach the cells completely, and then added DMEM medium to terminate the digestion. We transferred the cell suspension to a centrifuge tube for centrifugation at 1000 rpm for 5 min. Then, the medium was discarded and the cells were resuspended in PBS to make a cell suspension for washing, and the cells were transferred to a 1.5 mL centrifuge tube and centrifuged at 1000 rpm for 5 min to collect the cells, after which pre-cooled 70% ethanol was added and the cells were fixed at 4 °C for 1–4 h. After centrifugation and removing the fixing solution, the cells were resuspended in 1 mL of pre-cooled PBS. We then added 100 μL of PI staining solution to make a final concentration of 100 μg/mL, performed staining in the absence of light for 30 min, and analysed the cell cycle changes using flow cytometry.

### 4.11. H1N1 Influenza Virus Infects MDCK Cells

In this experiment, to study the effects of *THBS1* knockdown on influenza virus replication in CL23 cells and *THBS1* overexpression on influenza virus replication in M60 cells, we used well-grown, stably transfected *THBS1*-sh-con, *THBS1*-sh-122484, *THBS1*-OE-con, and *THBS1*-OE cells. They were grown in 6-well plates until they reached 90% confluence. Then, they were detached with 0.25% trypsin (Lanzhou Bailing, Lanzhou, China, CPT101.02) and counted, washed three times with PBS, and resuspended in serum-free DMEM medium. The number of cells was used to determine the volume of H1N1 virus fluid required to infect the cells in each group (MOI = 0.001). Then, we collected the virus infection supernatant, RNA samples, and protein samples at 36 h, 48 h, and 72 h, to determine the protein expression levels for the H1N1 influenza virus.

### 4.12. Statistical Analysis

All of the data used in this experiment are expressed as mean ± standard error of measurement (SEM) and were analysed using GraphPad Prism 8.0 software for paired samples t-test data variance analysis.

## Figures and Tables

**Table 1:** Primer sequences.

Gene Name	Primer Sequence (5′→3′)
Ataxin-1 (*ATXN1*)	F: CTGAAGAAGGTGGAGGACTTG
	R: CCGACGGCAAACTGTATCA
BCL2-Associated X (*Bax*)	F: TCCCCGTGAGGTCTTCTTCC
	R: GGGCCTTGAGCACCAGTTT
B-cell lymphoma-2 (*Bcl2*)	F: TCATCCAAGAATGCAAAGCAC
	R: CCCGGTTATCGTACCCTGTT
*Cyclin D1*	F: TGCCAGTGGCAGAGGAGAA
	R: TGGAGGGTGGGTTGGAAAT
EPH receptor B2 (*EPHB2*)	F: GTACCTGGCAGACATGAACTAC
	R: GAAAGCGTGAAAGCCCAAAG
Glyceraldehyde-3-phosphatedehydrogenase (*GAPDH*)	F: TGACGACATCAAGAAGGTAGTG
	R: AGTGGGTGTCACTGTTGAAG
Nucleoprotein (*NP*)	F: TGTCAGCTATTATGGAGCTGTTAG
	R: TGTCAGCTATTATGGAGCTGTTAG
Non-Structural Protein 1 (*NS1*)	F: CGAAATTTCACCATTGCCTT
	R: GTGGAGGTCTCCCATTCTCA
Scavenger Receptor Class B, Member 2 (*SCARB2*)	F: AAGAGCACCCCTCATACGAAC
	R: CTCCTCCCTCCTCACTACAACA
Mothers against decapentaplegic homolog 2 (*Smad2*)	F: AGACCTTCCACGCATCACA
	R: CACTATCACTTAGGCACTCAGCA
Mothers against decapentaplegic homolog 3 (*Smad3*)	F: TGACCACCAGATGAACCACA
	R: TCGCAGTAGGTGACAGGCT
Transforming growth factor-β1 (*TGF-β1*)	F: CTGCCCACCTGGAACCTACT
	R: TTCCTTCAAGTCCCAAATGCT
Thrombosponin-1 (*THBS1)*	F: CTATGACAAGGACGGGATTGG
	R: ATACTGGGCTGGGTTGTAATG
*TP53*	F: GTTGGCCTGCACAGTTGGT
	R: GTTGGCCTGCACAGTTGGT
Uroplakin3A (*UPK3A*)	F: GTGGCCTTTGGCCTGAT
	R: GAGGTACGCGTTCAGGATTT
*β-actin*	F: CTGCCCACCTGGAACCTACT
	R: TTCCTTCAAGTCCCAAATGCT

## Data Availability

Mandates data sharing and peer reviews data.
